# Pembrolizumab combined with lenvatinib and metronomic cyclophosphamide in platinum-resistant ovarian cancer: a case report of durable clinical response

**DOI:** 10.3389/fonc.2025.1582701

**Published:** 2025-08-20

**Authors:** Guanlin Dai, Furong Tang, Ping Wang, Danqing Wang

**Affiliations:** ^1^ Department of Obstetrics and Gynecology, West China Second University Hospital, Sichuan University, Chengdu, Sichuan, China; ^2^ Key Laboratory of Birth Defects and Related Diseases of Women and Children (Sichuan University), Ministry of Education, Chengdu, Sichuan, China

**Keywords:** platinum-resistance, ovarian cancer, immunotherapy, pembrolizumab, lenvatinib, case report

## Abstract

Most patients with ovarian cancer experience disease recurrence or progression, and ultimately progress to platinum resistance. Standard treatments for platinum-resistant ovarian cancer (PROC) include non-platinum chemotherapy, targeted agents, and immunotherapy. Despite recent advances in individualized management of PROC, median progression-free survival remains limited. Effective treatments are still lacking for PROC treatment. Given the current landscape of immunotherapy in ovarian cancer, research is ongoing to investigate immune modulators to counteract immune escape and enhance the efficacy of immune checkpoint inhibitors. Here, we reported a successful administration of a triple regimen comprising pembrolizumab, lenvatinib and metronomic cyclophosphamide, as the third-line treatment in a patient with PROC. This combination resulted in a durable response, with a PFS of 52 months as of the last follow up. This is the first report on this triple regimen in PROC and its promising outcome suggested that this regimen deserves further investigation as a potential therapeutic option for PROC.

## 1 Introduction

Epithelial ovarian cancer is the leading cause of gynecological cancer-related mortality. According to statistics, approximately 61,100 new cases and 32,600 deaths occur annually in China, making it the second most common gynecological malignancies (1). Epithelial ovarian cancer is characterized by its insidious onset, with over 75% of patients presenting with advanced disease at diagnosis (2). The combination of surgical intervention and platinum-based chemotherapy remains the standard treatment. Targeted agents, including antiangiogenic agents and poly ADP-ribose polymerase (PARP) inhibitors, have been the first-line treatments for ovarian cancer, which can effectively extend patients’ survival (3). However, more than 70% of patients recur within three years, and approximately 50% eventually progress to platinum-resistance (3, 4). Platinum-resistant ovarian cancer (PROC) often implies constrained treatment options, suboptimal efficacy, and poor prognosis. Current standard treatments for PROC mainly include non-platinum chemotherapy including weekly paclitaxel, pegylated liposomal doxorubicin (PLD), or topotecan, which can be used alone or in combination with bevacizumab (5). However, the response rate of this regimen is relatively low, at approximately 10% to 15%, and the duration of response is only 3 to 4 months, with a median overall survival (OS) of around 12 months (5–9). The addition of bevacizumab, or other combined regimes, while beneficial, cannot significantly extend survival (5). Additionally, the cumulative toxicity associated with chemotherapy may also impede the continuation of treatment. Mirvetuximab soravtansine (MIRV) is an antibody-drug conjugate targeting folate receptor alpha (FOLRα). MIRV monotherapy has demonstrated promising antitumor activity in patients with PROC and is FDA-approved for the treatment of only FOLRα-positive subset (10). Approximately 35-40% of epithelial ovarian cancer shows FOLRα overexpression and may benefit from MIRV (11, 12). However, treatment option remains limited for patients with PROC who are ineligible for this treatment. The clinical need in PROC is still unmet.

Immunotherapy, particularly immune checkpoint inhibitors (ICIs), has achieved breakthrough progress in multiple solid tumors, and now have been approved for several malignancies. However, epithelial ovarian cancer exhibits limited response to immunotherapy, primarily due to its inherent immunosuppressive within tumor microenvironment (TME). Studies indicate that only 30% of ovarian cancer express programmed death receptor-1 (PD-1) or its ligand (PD-L1), and the response rate to ICI monotherapy remains modest, around 10% (13, 14). Currently, research on ICIs is focused on investigating immune modulators to counteract immune escape, alleviate immunosuppression within TME and amplify the efficacy of ICIs.

The combination of antiangiogenic agents, such as bevacizumab, with ICIs has demonstrated a synergistic effect, and this combination has shown significant efficacy in various solid tumors. Lenvatinib, a small-molecular tyrosine kinase inhibitor (TKI), exerts anti-tumor activity through targeting vascular endothelial growth factor receptors (VEGF 1-3), fibroblast growth factor receptors (FGFR 1-4), platelet-derived growth factor receptor-β (PDGFR), rearranged during transfection (RET), and stem cell factor receptor (15). In gynecological malignancies, lenvatinib has been primarily investigated in endometrial cancer (16, 17). The combination of lenvatinib and pembrolizumab has achieved substantial benefit, leading to its FDA approval in 2019 for the treatment of advanced or recurrent endometrial cancer, excluding MSI-H or dMMR subtype (18). However, its clinical evaluation in ovarian cancer remains limited and the Leap-005 trial is the only study to date assessing the anti-tumor activity of this combination in recurrent ovarian cancer (19).

Metronomic chemotherapy is defined as frequent, low-dose chemotherapy, which enhances anti-tumor activity of antiangiogenic agents with minimal toxicity and participants in immune modulation (20). In this study, we administrated a combination of lenvatinib, pembrolizumab and metronomic cyclophosphamide as the third line treatment in a patient with PROC. At the time of writing, this patient has achieved durable response with a progression-free survival (PFS) of 52 months. This is the first report on this triple regimen in PROC.

## 2 Case description

A 43-year-old woman presented to our hospital in November 2018 with a palpable pelvic mass persisting for a week. Contrast-enhanced computer tomography (CT) revealed a right-adnexal mass measuring approximately 35 x 50 mm with irregular enhancement, along with multiple peritoneal nodules. The excisional biopsy of mass confirmed malignancy, and this patient subsequently underwent cytoreductive surgery. Histopathological examination established a diagnosis of high-grade serous adenocarcinoma, staged IIIC. Postoperatively, she started platinum-based chemotherapy for six cycles, and the last course completed in April 2019. A follow-up CT scan demonstrated complete remission, and tumor biomarkers normalized. The patient followed up regularly thereafter.

The first recurrence occurred in November 2019, with a disease-free interval of seven months. During follow-up, a repeat CT scan showed tumor recurrence involving peritoneum and liver capsule, accompanied by an elevation in CA125 levels to 16.3 U/ml. This patient was a platinum-sensitive recurrence but this time, she was not considered a candidate for secondary cytoreductive surgery though evaluation by gynecologic oncologists. Consequently, she underwent the second-line chemotherapy with cisplatin and paclitaxel plus bevacizumab for six cycles, completing the treatment in March 2020. During the treatment, this patient experienced severe adverse effects (AEs) including myelosuppression, hypertension, vomiting, and alopecia. A post-treatment CT scan showed partial response, with regression of peritoneal and liver capsule nodules. CA125 levels decreased to the normal range. Upon discharge, maintenance therapy with niraparib (200 mg orally daily) was initiated based on NCCN guidelines (2020.v1).

**Figure 1:**
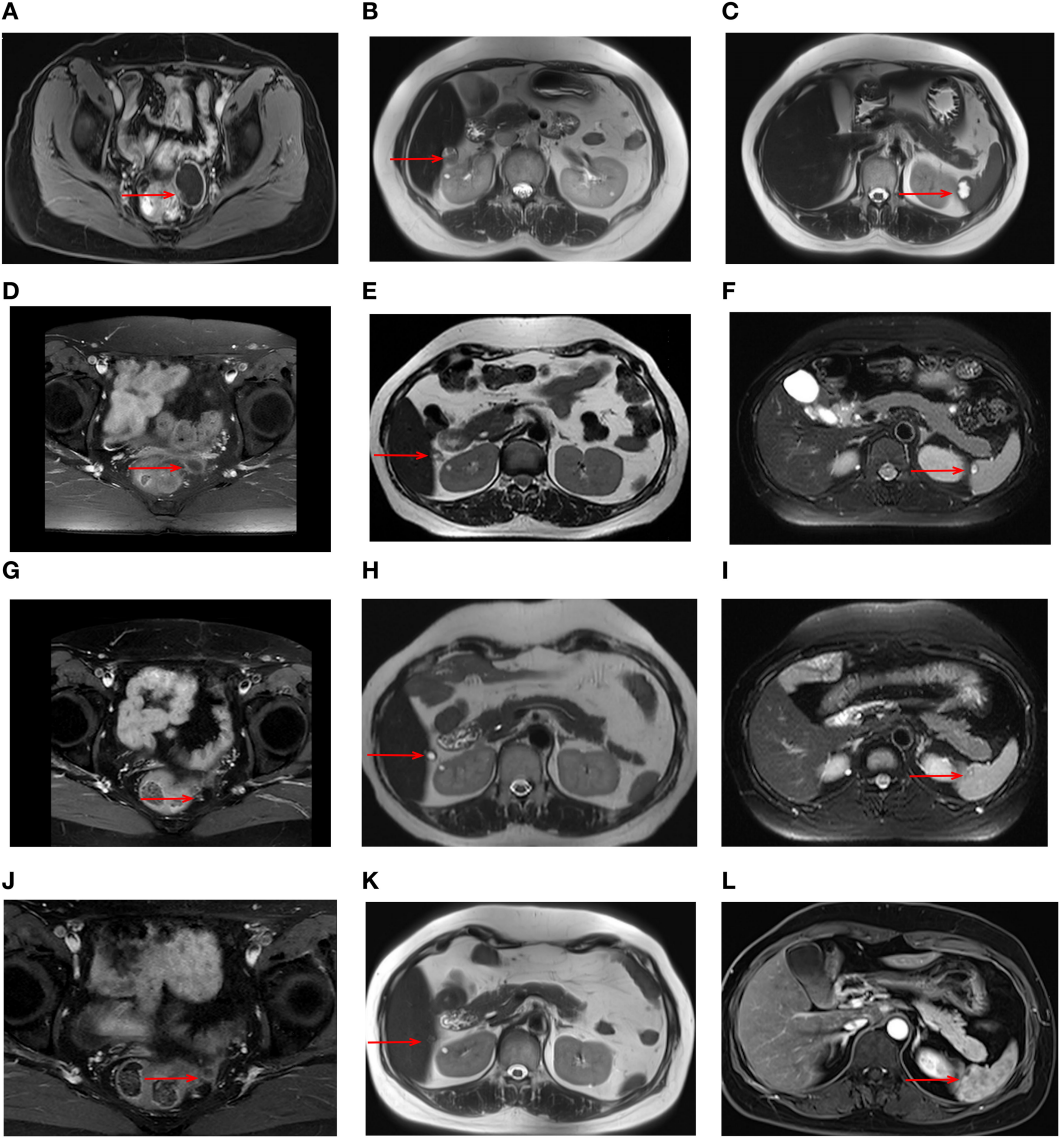
MRI images during treatment with the triple regimen. MRI images of the tumors (red arrows) are shown at different stages; Prior to the treatment: pelvic mass **(A)** liver capsule **(B)** spleen **(C)** After 6 cycles: pelvic mass **(D)** liver capsule **(E)** spleen **(F)** After 22 cycles: pelvic mass **(G)** liver capsule **(H)** spleen **(I)** After 33 cycles: pelvic mass **(J)** liver capsule **(K)** spleen **(L)**.

During maintenance therapy, tumor biomarkers, including CA125, showed a gradual increase. After three months, CA125 levels increased to 28.5U/ml. Magnetic resonance imaging (MRI) in July 2020 showed a cystic-solid mass measuring 42 x 33 mm in the pelvis along with significant progression of the liver capsule and new involvement of spleen (**Figures 1A–C**). Given these findings, disease progression was diagnosed, but this time, the gynecologic oncologist considered a platinum-resistant recurrence since this progression occurred on maintenance therapy. In accordance with the latest NCCN guideline, non-platinum chemotherapy with or without bevacizumab is recommended. While this patient adamantly declined the recommended therapy due to severe AEs experienced during prior chemotherapies.

**Figure 2:**
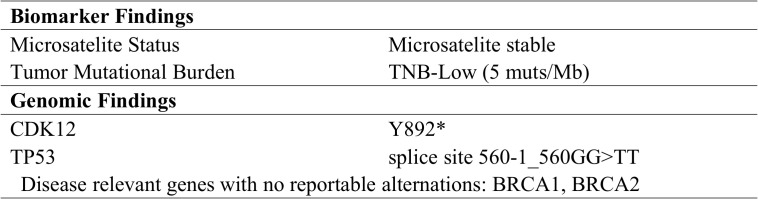
Details of genomic and biomarker findings.

**Figure 3:**
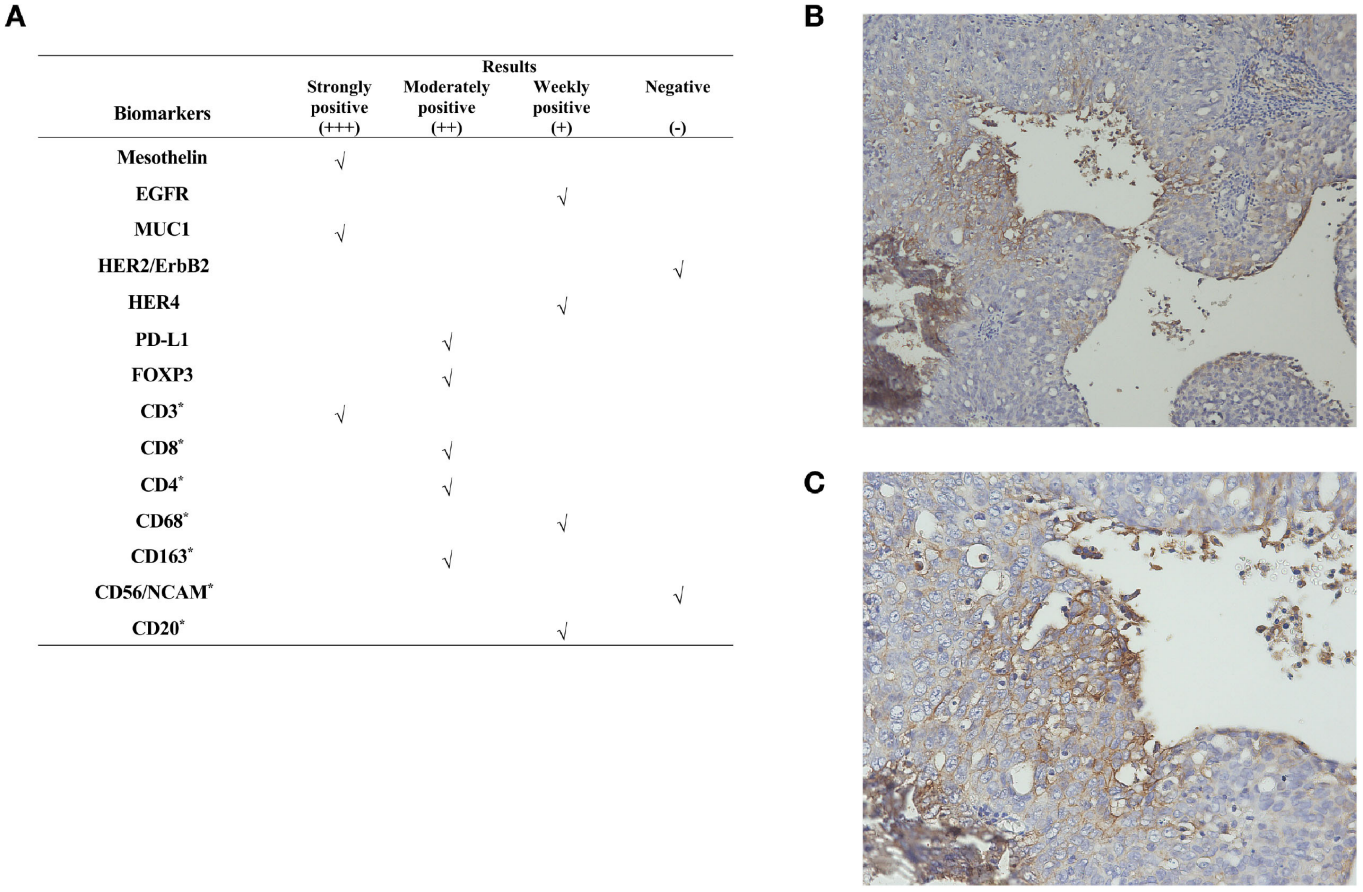
Immunohistochemical staining results. **(A)** Immunogenomic profile of the tumor. Representative images of IHC for PD-L1 expression at **(B)** ×100 and **(C)** ×200 magnification. “*”represents a marker for immune cells. **The CPS is defined as the number of PD-L1–staining cells including tumor cells, lymphocytes, and macrophages, divided by the total number of viable tumor cells, multiplied by 100. A CPS of ≥1 was considered positive for PD-L1 expression.

To explore alternative therapeutic regimens, we reviewed the genetic testing which included genes that were validated targets for therapy, either approved or under clinical trial investigation, and that are unambiguous driver of oncogenesis based on current knowledge. Genomic findings revealed the only somatic mutations of TP53 and CDK12, and disease relevant genes including BRCA1 and BRCA2 showed no reportable alternations. The biomarker findings revealed microstatellite stable and tumor mutational burden was low of 5 muts/MB (**Figure 2**). Immunohistochemical staining of the tumor tissue showed the moderate expression of PD-L1 in tumor lesions, with the combined positive score of 3 (**Figures 3A–C**).

**Figure 4:**
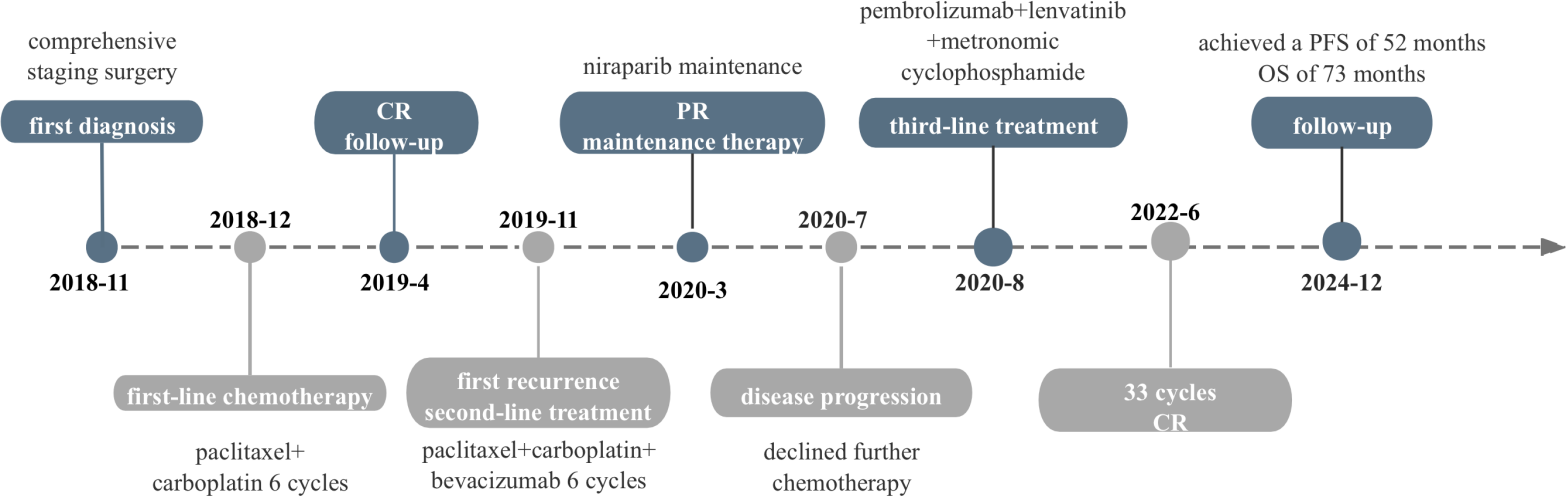
Timeline of the diagnostic and treatment process.

At that time, immunotherapy represents a viable treatment option for this patient. Given the limited responsiveness of ovarian cancer to immunotherapy, we comprehensively evaluated both the efficacy and mechanisms of the reported combination regimens. Following a multidisciplinary discussion and obtaining informed consent from the patient and her relatives, this patient started combination immunotherapy on August 2020, comprising pembrolizumab (200mg intravenously every three weeks), lenvatinib (12 mg orally everyday) and metronomic cyclophosphamide (50mg orally every day). During treatment, the patient underwent clinical evaluations every 3–6 treatment cycles, including physical examinations, laboratory tests, and imaging assessments, with treatment response evaluated according to the RECIST 1.1 criteria. Imaging evaluations included both CT and MRI. In this case, the patient subsequently opted for long-term MRI monitoring due to a contrast-induced allergic reaction developed during the last imaging evaluation. After 6 cycles, serum CA125 levels normalized, and follow-up MRI showed a significant response to this combination treatment (**Figures 1D–F**). By the 22nd cycles, radiological assessment confirmed partial remission (**Figures 1G–I**). Due to the development of severe hypertension, lenvatinib was reduced to 8 mg once daily. Thereafter, the patient continued the combination regimen with mild adverse effects, achieving a sustained clinical response for over 50 months (**Figures 1J–L**). At the time of this writing, this patient has achieved an OS of 73 month and PFS of 52 months. As of the latest follow-up in December 2024, the disease remains stable without signs of recurrence. The timeline of the diagnostic and treatment process is listed in **Figure 4**.

## 3 Discussion

The application of PD-1/PD-L1 inhibitors as single-agent treatment for ovarian cancer is mostly in phase I–II clinical trials. For patients with advanced and recurrent ovarian cancer, the ORRs are generally around 10% (13, 14). Compared with other tumors, PD-1/PD-L1 inhibitors demonstrate efficacy only in a small subset of ovarian cancer. However, patients with PROC commonly imply limited treatment options, and poorly response to non-platinum chemotherapy. The cumulative toxicity of prior chemotherapies often leads to treatment suspending. Moreover, in some cases, no other actionable molecular targets were detectable. Research on immunotherapy for ovarian cancer is still persistently exploring, and recently, research mainly focuses on combining immunotherapy with other therapeutic modalities to treat refractory and recurrent ovarian cancer. Previous studies have shown that chemotherapy can augment the self-immune activation. In clinical trials, compared with the single-agent PD-1/PD-L1 inhibitors, the combinations with chemotherapy have a slight improvement in ORR but with modest response duration. The JAVELIN Ovarian 200 (NCT02580058) trial evaluated the antitumor activity of avelumab, PLD as monotherapy, and their combination in PROC. The results did not show a significant benefit of the combination therapy over PLD monotherapy, with the OS of 15.7 months and 13.1 months, respectively, and PFS of 3.7 months and 3.5 months (21).

The study of the combination of PD-1/PD-L1 inhibitors with targeted agents are also in progress, of which the overall ORRs of the combination with anti-angiogenic agents ranges from 15 to 33%. The phase Ib clinical trial evaluated the efficacy of atezolizumab in combination with bevacizumab in 20 patients with PROC (22). The results showed an ORR of 15% and disease control rate was 55%. The median PFS was 4.9 months (range, 1.2-20.2 months), and the median OS was 10.2 months (range, 1.2-26.6 months) (22). In the phase II clinical trial of the combination of nivolumab and bevacizumab (NCT02873962), the results showed an overall ORR of 28.9%, with an ORR of 40% in the platinum-sensitive cohort and 16.7% in PROC. The overall clinical benefit rate reached 55.3%, with 75% of platinum-sensitive patients and 33.3% of PROC (23). In the Phase II clinical trial LEAP-005 (NCT03797326) of the combination of pembrolizumab and lenvatinib, among 31 patients with metastatic or unresectable ovarian cancer, the ORR was 32%, the rate of disease control was 74%, and the median PFS was 4.4 months (range, 4-8.5 months) (19). The phase II clinical trial NCT02853318 incorporated metronomic cytotoxic chemotherapy into the combination of pembrolizumab and bevacizumab. The overall ORR reached 47.5% (24). In PROC subgroup, the ORR reached 43.3% (90% CI: 29.6%-58.2%), with a median PFS of 7.6 months (95% CI: 5.7-10.0 months). Compared to the dual combination treatment of PD-1/PD-L1 inhibitors and antiangiogenic agents, this triplet regime demonstrated superior efficacy particularly in PROC. Based on this trial (NCT02853318), the 2024 NCCN guidelines for ovarian cancer have included this triple regimen as a recommend option for PROC (24). In terms of the combination with PARP inhibitors, in the MEDIOLA study, olaparib combined with durvalumab were administrated to patients with germline BRCA mutations, the ORR reached 71.9% in platinum-sensitive disease (25). However, in the TOPACIO/KEYNOTE-162 study, niraparib combined with pembrolizumab was administrated to patients with PROC, the ORR was only 18% (26). More research and verification are needed in the combination immunotherapy with PARP inhibitors in PROC.

The integration of PD-1/PD-L1 inhibitors with antiangiogenic agents has demonstrated superior efficacy in multiple solid malignancies, especially the combination of pembrolizumab and lenvatinib has made a sustainable advancement in the management of advanced endometrial cancer. Abnormal angiogenesis is a distinctive feature of solid tumors and is involved in tumor immune evasion. Antiangiogenic agents primarily work by blocking the active VEGF/VEGFR signaling pathways in the TME under hypoxic conditions. They not only normalize tumor vasculature but also reverse the VEGF-mediated immunosuppression. On the one hand, antiangiogenic agents facilitate antigen presentation and enhance the activation of CD8+ T cell to stimulate immune responses. Mature dendritic cells (DCs) are negatively correlated with VEGF levels, and immature DCs cannot present cancer antigens to T cells. Antiangiogenic agents can relieve the inhibitory effects of VEGF on DCs and stimulate antigen presentation (27). On the other hand, antiangiogenic agents suppress the apoptosis of CD8+ T cells through inhibiting the expression of PD-1. Antiangiogenic agents also can facilitate the infiltration and migration of lymphocytes, as the migration of lymphocytes from the bloodstream into the tumor stroma is affected by the integrity of the tumor vasculature (27). Additionally, antiangiogenic agents reprogram the tumor vasculature, promoting vascular normalization and reducing the immunosuppressive effects of Tregs, MDSCs, and TAMs. ICIs mainly exert their antitumor effects by relieving the functional suppression of T cells by tumor cells within TME (28). Antiangiogenic therapy and immunotherapy both act on the TME, influencing each other and working synergistically to enhance efficacy.

Unlike bevacizumab that only focuses on the VEGF/VEGFR signal pathway, lenvatinib is a multi-targeted TKI, which not only targets the VEGF/VEGFR signaling pathways but also inhibits the FGFR/FGFR, PDGF/PDGFR, and RET signaling pathways. The blocking of the additional signal pathways plays a critical role in the reformation of the TME which mainly manifestations include significantly increased CD8+ T-cell infiltration, reduced inhibitory immune cells (such as T-regs, TAMs), and upregulated PD-L1 expression (29, 30). The broader spectrum of inhibition can enhance the antitumor activity and the impact of immunomodulation synergistically with pembrolizumab.

Metronomic cyclophosphamide refers to the frequent low-dose cyclophosphamide. It can enhance the anti-tumor activity of antiangiogenic agents through blocking VEGFs. The combination of bevacizumab with metronomic cyclophosphamide has demonstrated superior antitumor activity compared to bevacizumab monotherapy in clinical trial (20). Beyond its anti-angiogenic properties, metronomic cyclophosphamide selectively reduces the quantity and activity of regulatory T cells, thereby alleviating immunosuppression within the TME and restoring the function of cytotoxic T cells and natural killer cells (20, 24). Although metronomic cyclophosphamide primarily exerts the anti-tumor activity through immune modulation and anti-angiogenesis, continuous low-dose administration accumulates drug concentration within the TME, enabling direct cytotoxic effects on tumor cells (20).

In this report, the patient experienced platinum-resistance after second-line chemotherapy. At that time, NCCN guideline recommended the preferred regime including non-platinum chemotherapy with or without bevacizumab was not feasible. On the one hand, the response rate of this traditional regimen is relatively low, and the effect is not satisfactory. On the other hand, the adverse reactions of this regime are serious, and the patient was in poor condition with severe adverse reactions attributed to prior-lines chemotherapy. The patient and her relatives adamantly refused this regime after they were informed. The genetic testing, which included genes that are validated targets for therapy, either approved or under clinical trial investigation, revealed only somatic mutations in TP53 and CDK12, with no other actionable targets identified. The biomarker findings showed only positive expression of PD-L1, without other actionable targets such as FLORα expression, which could benefit from MIRV. At that time, combination immunotherapy represents a viable treatment option for this patient. Given the limited responsiveness of ovarian cancer to immunotherapy, we comprehensively evaluated the efficacy of the reported combination regimens and mechanisms of drug interaction. Following a multidisciplinary discussion and obtaining informed consent from the patient and her relatives, this patient initiated on a combination regimen of pembrolizumab (200mg intravenously every three weeks), lenvatinib (12 mg orally everyday), and metronomic cyclophosphamide (50mg orally every day). Both serum biomarkers and MRI could indicate a significant response following 6 cycles. During treatment, the dose of lenvatinib reduced to 8 mg daily due to the development of severe hypertension. Nevertheless, she continued this combination and achieved a durable response for more than 50 months with tolerable side effects.

Despite remarkable antitumor activity of lenvatinib in combination with pembrolizumab, it is also associated with increased toxicity, primarily attributed to lenvatinib. Previous research indicated that the incidence of hypothyroidism in combined administration of pembrolizumab and lenvatinib is higher than that in monotherapy with lenvatinib, with an incidence rate of 57.4%, compared to 47.2% for monotherapy (31). While the incidence of grade 3 or higher immune-related AEs is relatively low, ranging from 0.9% to 1% (31). The most common AEs include gastrointestinal reaction, hepatotoxicity, thyroid dysfunction and hypertension, frequently necessitating dose reductions or discontinuation. The KEYNOTE-775 trial in advanced endometrial cancer confirmed this burden that 66.5% of patients on standard-dose lenvatinib (20 mg) required dose reductions, and 30.8% discontinued lenvatinib due to adverse events (32). How et al. suggested that starting lenvatinib at a reduced dose (14 mg) in recurrent endometrial cancer significantly decreased the need for subsequent reductions and delayed drug-related toxicity development compared to the standard dose (20 mg), without compromising ORRs (33). This underscores the potential for optimized dosing to manage toxicity while preserving efficacy. Proactive toxicity management, and dose individualization are crucial in this combination.

As a single-patient observation, while promising it carries the risk of selection bias, lacks generalizability and statistical power. Uncontrolled factors including the patient’s molecular profile and intervention timing may be contributed to this unexpected outcome which may not be representative of all patients with PROC. In addition, the retrospective analysis limited us to perform comprehensive biomarker analyses, such as dynamic assessment of TME evolution during treatment. These factors preclude the generalizability of this triple treatment to broader PROC populations. Future research will require large-scale, randomized, multi-center clinical trials to validate the antitumor activity of this combination regime in PROC, and to better understand the potential benefits and risks of this triple treatment regimen. Additionally, further research should focus on identifying specific patient populations that are most likely to benefit from this combination therapy, as well as exploring the underlying mechanisms that contribute to the observed clinical response.

## 4 Conclusion

This is the first report documenting the efficacy of the triple regimen combining pembrolizumab, low dose lenvatinib, and metronomic cyclophosphamide in a patient with PROC. The prolonged response and superior PFS observed in this case suggest that this triple regimen may serve as a promising therapeutic option for PROC. Future studies involving larger cohorts are needed to validate these results and to better understand the potential benefits and risks of this triple treatment regimen. Finally, on the basis of standard treatment, personalized precision therapy is the direction of cancer treatment. Assessing, classifying, and selecting individualized treatment regimens based on different individuals is an important direction for future research.

## Data Availability

The original contributions presented in the study are included in the article/supplementary material. Further inquiries can be directed to the corresponding author.
